# Understanding visual consciousness in autism spectrum disorders

**DOI:** 10.3389/fnhum.2015.00204

**Published:** 2015-04-21

**Authors:** Tal Yatziv, Hilla Jacobson

**Affiliations:** ^1^Department of Psychology, Ben-Gurion University of the NegevBeer-Sheva, Israel; ^2^Zlotowski Center for Neuroscience, Ben-Gurion University of the NegevBeer-Sheva, Israel; ^3^Department of Brain and Cognition Sciences, Ben-Gurion University of the NegevBeer-Sheva, Israel

**Keywords:** autism spectrum disorders, phenomenal consciousness, access consciousness, perceptual integration, categorization

## Abstract

The paper focuses on the question of what the (visual) perceptual differences are between individuals with autism spectrum disorders (ASD) and typically developing (TD) individuals. We argue against the view that autistic subjects have a deficiency in the most basic form of perceptual consciousness—namely, phenomenal consciousness. Instead, we maintain, the perceptual atypicality of individuals with autism is of a more conceptual and cognitive sort—their perceptual experiences share crucial aspects with TD individuals. Our starting point is Ben Shalom’s (2005, 2009) three-level processing framework for explaining atypicality in several domains of processing among autistics, which we compare with two other tripartite models of perception—Jackendoff’s (1987) and Prinz’s (2000, 2005a, 2007) Intermediate Level Hypothesis and Lamme’s (2004, 2006, 2010) neural account of consciousness. According to these models, whereas the second level of processing is concerned with viewer-centered visual representations of basic visual properties and incorporates some early forms of integration, the third level is more cognitive and conceptual. We argue that the data suggest that the atypicality in autism is restricted mainly to the third level. More specifically, second-level integration, which is the mark of phenomenal consciousness, is typical, yet third-level integration of perceptual objects and concepts is atypical. Thus, the basic experiences of individuals with autism are likely to be similar to typical subjects’ experiences; the main difference lies in the sort of cognitive access the subjects have to their experiences. We conclude by discussing implications of the suggested analysis of experience in autism for conceptions of phenomenal consciousness.

What are the (visual) perceptual differences between individuals with autism spectrum disorders (ASD) and typically developing (TD) individuals? Do autistic subjects have a deficiency in what is called in standard philosophical jargon “phenomenal consciousness”—the most basic form of perceptual consciousness? Or is it that the perceptual difference between individuals with ASD and TD individuals is of a more conceptual and cognitive sort, whereas the most basic perceptual experiences of individuals belonging to these two groups are similar? Ben Shalom (2005, 2009) suggests a three-level processing framework to explain atypicality in several domains of processing among individuals with ASD, which is highly relevant to these questions. She argues that individuals with autism and TD individuals do differ in their basic perceptual experiences. In what follows we will discuss the (visual) perceptual differences between individuals with ASD and TD individuals, taking Ben Shalom’s tripartite model of perception as a starting point, and present our own view on the issues at hand. We will argue that the evidence tells against the view that autistic subjects have a deficiency in basic perceptual consciousness and support the hypothesis that the perceptual difference between autistics and TD individuals is of a more conceptual and cognitive sort.

In the first section we examine the three-level distinction of perceptual processing proposed by Ben Shalom, by comparing it with two other tripartite models of perceptual processing—Jackendoff’s and Prinz’ Intermediate Level Hypothesis (Jackendoff, 1987; Prinz, 2000, 2005a, 2007) and Lamme’s (2004, 2006, 2010) neural account of consciousness—while emphasizing the role of integration in each model. In the second section we introduce the debate regarding the possible dissociation between two types of consciousness: phenomenal consciousness (or experience) and access consciousness. We discuss each model’s stance with regard to this debate. In the third section, we argue that the evidence suggests that in autism second-level integration, which is the mark of phenomenality, is typical, yet third-level integration of perceptual objects and concepts is atypical. In the fourth section we further clarify that argument, and show, further, that it supports the view that individuals with ASD and TD individuals do not differ in their basic experiences, but rather have different cognitive access to those experiences. We conclude by discussing implications of the suggested analysis of experience in autism for conceptions of phenomenal consciousness.

## Levels of Perceptual Processing: Examination of Ben Shalom’s Model

### Tripartite Models of Perceptual Processing

There are several tripartite models of perceptual processing in the literature on perceptual consciousness. Here, we discuss similarities and differences between three such conceptualizations. The first is Ben Shalom’s (2005, 2009) framework, used in her analysis of perceptual atypicality in ASD. The other two models are Jackendoff’s and Prinz’ Intermediate-Level Hypothesis (henceforth, ILH; Jackendoff, 1987; Prinz, 2000, 2005a, 2007), and Lamme’s (2004, 2006, 2010) neural account of perceptual consciousness, both of which pertain to the explanation of perceptual experience.

According to Ben Shalom (2005, 2009), processing in different psychological domains takes place at three general levels of processing: basic, integrative and logical. Specifically, in the perceptual domain, conscious object recognition is suggested to involve processing in three levels: (1) a level of basic processing, which does not include a conscious experience of the percept; (2) an integrative level based on perceptual objects, which includes experience; and (3) a logical higher-order level of processing. Ben Shalom suggests that the phenomenon of perceptual object fragmentation among individuals with ASD is the result of atypicality at the integrative level, due to lack of prioritization of the whole integrated object at the expense of object features. The reduced salience of the integrated object can be overcome by effortful logical processing of perception at the third level.

The ILH, proposed by Jackendoff (1987) and elaborated on recently by Prinz (2000, 2005a, 2007), claims that visual processing takes place at three stages that vary in their abstraction, and that conscious visual experience arises at the intermediate level. Similarly to Ben Shalom’s model, the theory can be applied to several domains, and here we focus on perception. In the visual modality, the ILH is inspired by Marr’s (1982) theory of visual processing. The low-level is “the primal sketch level”, which is organized retinotopically, and is responsible for encoding blobs, bars and edges. This level is highly detailed, but does not include a coherent representation of an object. The intermediate-level is “the 2.5D sketch level”, which is responsible for the creation of a coherent representation of the object from the specific viewpoint of the observer. The high-level—the “3D model level”—contains a viewpoint-invariant representation that lacks specific details, allowing for the assignment of the object to an abstract category. According to both Prinz and Jackendoff, the high level is ideal for object recognition. That is, the perceptual hierarchy starts from the registration of specific local features, proceeds to the combination of these features into coherent objects (e.g., feature binding) and ends with the processing of abstract properties of the object. Based on introspective and empirical arguments, Jackendoff and Prinz claim that only intermediate-level representations match the content of perceptual consciousness: representations of coherent bound-together objects, including their features represented from a specific point of view.

Lamme (2004, 2006, 2010) distinguishes among three levels of perceptual processing, based on neural processing characteristics: (1) a low level of fast feedforward sweeps; (2) a level of superficial recurrent processing that is limited to visual areas; and (3) a level of deep recurrent processing, in which recurrent processing becomes widespread. At the first level, feedforward sweeps flow serially throughout the visual stream, enabling feature extraction.^1^ At the second level—the superficial recurrent processing level—each area to which the feedforward sweeps reaches begins sending feedback to lower levels in local groups, thus enabling perceptual integration of different aspects of objects and scenes into a coherent percept. Similarly to ILH’s conception of the intermediate-level, the superficial recurrent processing level is characterized as similar to Marr’s 2.5 sketch (Lamme, 2004) and as neural activity that gives rise to perceptual experience. The third level—the deep recurrent processing level—involves attentional selection of a limited number of local recurrent processes, and manifests in the amplification of these processes to widespread co-activation of visual and frontoparietal regions. Widespread recurrent processing allows accessibility of attended objects to other processes. This stage corresponds to the global workspace conception (Dehaene and Naccache, 2001; Baars, 2005; Dehaene et al., 2006) and to working memory (WM). It should be noted that Lamme’s model is highly consistent with other recent accounts of simultaneous bottom-up and top-down processing (e.g., Hochstein and Ahissar, 2002; Bar et al., 2006), which influenced Ben Shalom’s model as well (Ben Shalom, 2005, 2009).

### The Importance of Integration for Experience

It is noticeable that the three models outlined above share some common ground. Overall, the three models seem to share the same conceptualization of perceptual processing. In all cases, processing begins with extraction of individual features, proceeds with integration of features into coherent objects and ends with abstraction and reasoning. Moreover, all three seem to agree that the content of experience is achieved at the second level of the hierarchy. Both Lamme and Jackendoff and Prinz explicitly argue for this claim and we will shortly outline their considerations in its favor. Ben Shalom does not explicitly present the reasons for her claim that experience should be ascribed at the integrative level. However, her analysis seems to imply that the integration of features into a coherent object is necessary for the emergence of experience.

The main goal of ILH is to claim that the 2.5 sketch level is the locale of the content of experience (Prinz, 2007), i.e., that the content of experience is constituted by second-level representations. According to Jackendoff (1987), the locale of the content of conscious experience can be derived from introspection: the content of our experience is neither as specific as low-level representations nor as abstract as high-level representations, but is rather revealed to us as a coherent bound-together object, including its features represented from a specific point of view. A similar point is vividly made by Treisman (2003): when trying to imagine a triangle, we picture a very specific one, with a certain shape (e.g., a right angle triangle), size, length of sides, orientation, color and location in space, so that all these features are bound together in a particular way. Beyond the argument from introspection, Prinz (2000, 2005a, 2007) argues that detailed viewpoint-specific representations are necessary for deliberate behavioral responses, and that patterns of neuropsychological damage after lesions in areas related to each level support this conclusion as well. However, it should be noted that according to Prinz (2011a), as part of his AIR theory (attended intermediate-level representations theory), the mere processing of representations in the intermediate level is not sufficient for consciousness—only stimuli whose representations are modulated by attention and made accessible to WM are experienced. That is, although the second level is the locale of the *content* of experience, not every second-level representation is experienced.

Lamme (2010) has not only argued in favor of localizing the content of experience at the second level, but also in favor of localizing experience itself at this level. He has argued that representations processed at the second level are phenomenally conscious as part of his neural argument, an argument that bears on the debate that we shall soon present. The neural argument is based on several assumptions. First, Lamme assumes that first-level representations are not experienced. This seems to be agreed on by all models, and consistent with introspection. The second assumption, which is also reasonable and uncontentious, is that third-level representations are conscious, since they are accessible for use in reasoning and behavioral control through WM or the global workspace (i.e., available for report, reasoning and the production of intentional behavior). The third assumption, which is the most important to our current discussion, is that neural processing at the second level is more similar to the third level than to the first, since it underlies properties necessary for consciousness, notably integration and plasticity. Recurrent processing, whether deep or superficial, allows interactions between areas, so that processing in areas that are located higher in the feedforward hierarchy affect and modify processing in lower areas of the hierarchy. This enables aspects of integration such as feature binding and grouping, which, as argued above, are key properties of experience. As Lamme emphasizes, second level activity produces high Φ, which according to Tononi (2007) denotes the amount of integrated information generated by a system when switching from one processing state to another. In Tononi’s words, “subjective experience *is* integrated information” (2007, p.297; emphasis in the original), and generation of high Φ is the criteria for conscious systems. Recurrent processing also gives rise to plasticity and learning, by satisfying Hebb’s rule. Because the second level is more similar to the third level than to the first, Lamme concludes that second level representations are experienced as well. That is, according to Lamme the second level is not only the locale of the *content* of experience, but also the locale of experience* itself*: second-level representations are experienced in virtue of being processed at this level.

Although all three models agree that integration takes place at the second level, and that integration is essential for experience, they seem to disagree on the type of integration achieved at this level. According to Ben Shalom, integration at the second level is not only perceptual, but also includes precise categorization. For example, when perceiving a white plate, the integrative level is in charge of making the representation of the integrated object “plate” more prominent than the representations of its separate features, “white” and “round”. Due to abnormal integration, for people with ASD the representation of the object (e.g., “plate”) is not amplified (Ben Shalom, 2005).

In contrast, ILH suggests that precise categorization occurs only at the third level Prinz (2005a, 2007). The second level contains a 2.5D sketch of the world, and thus integration is interpreted as binding of features into a coherent object from a specific point of view. ILH argues that the third level is the one most suitable for object recognition, since the 3D model of the world contains abstract and viewpoint-invariant representations. As part of his defense on concept empiricism, Prinz has argued that concepts are mental representation of categories in WM (or that can be in WM), that are based on the identification of perceived objects (Prinz, 2002, 2005b, 2012; see also Barsalou, 1987, 2005). Concepts are constructed from representations in long-term memory that are based on third-level representation rather than on second-level representations. This entails that a representation of an object must be formed prior to categorization, at least in a tentative manner. Although this issue is debated in the philosophical literature, Prinz’s (2007, 2011a) stance is that high-level perceptual representations (such as concepts or categories, e.g., being a chair) are not part of the content of our experience: even-though third-level representations of concepts can be activated in WM, they are not experienced (but see, e.g., Siegel, 2010, for a contrary view). Prinz (2011b) supports this claim with an example of patients with semantic dementia, who cannot recognize objects and use conceptual knowledge but nonetheless have typical object perception phenomenology (e.g., match images). Thus, ILH would argue that when perceiving a white plate, the features “white” and “round” are bound together at the intermediate level, forming a combined representation of the conjunction “white and round”, whereas the category “plate” is assigned to the object at the third level, after the creation of a 3D viewpoint-invariant representation. It should be noted that this process is not strictly bottom-up, and top-down information can affect categorization as well as experience. For example, context can affect the concept representation that will be used in WM in a certain situation (Prinz, 2002, 2011b). However, in the absence of contextual information, a default representation of the concept will be active in WM. In addition, concepts can alter second-level representations in a top-down process.

In Lamme’s model, categorization is achieved gradually, with different degrees of categorization occurring at different levels. Although Lamme (2010) regards his second level as similar to Marr’s 2.5D sketch (2004), he also argues that conscious percepts are characterized by integration of features and categories, which enables feature-invariant processing and differentiation of the object from other categories (e.g., not a house), a description that better matches the 3D sketch. Lamme has suggested that there is a higher type of integration at the third level. For example, an identification of the stimulus as a face (rather than a still object) is achieved prior to recognition of the identity of the face (Lamme, 2010). Therefore, he may regard basic categorization (e.g., faces vs. still objects) as occurring at the second level and specific categorization (e.g., recognition of the type of still object as a plate) as achieved at the third level.

Lamme’s notion of degrees of categorization is in line with other accounts of categorization. According to the Two-State Interactive (2SI) account of visual object recognition proposed by Schendan and colleagues (Schendan and Kutas, 2007; Schendan and Stern, 2008), initial classification is achieved during fast feedforward processing, but precise categorization occurs later on, during co-activation and combined feedforward and feedback interactions between ventral-path areas and areas in the prefrontal cortex and medial temporal lobe. In this later stage, bottom-up and top-down information is integrated. Although there are some discrepancies between Lamme’s account and the 2SI account, they share the idea, which is important to our purposes, that precise categorization occurs at the third level. This idea is also consistent with Bar et al.’s (2006) evidence for early categorization of classes of objects according to low spatial frequencies, later used for top-down facilitation of object recognition through narrowing down of interpretation options (similar ideas are portrayed in the reverse hierarchy theory, see Hochstein and Ahissar, 2002). However, Bar’s model would predict earlier categorization during rapid feedback modulation (Bar, 2003; Bar et al., 2006; Schendan and Stern, 2008). Additionally, the 2SI theory fits well with Prinz’ suggestion that context can affect categorization in a top-down manner. Thus, there are reasons for thinking that for complex objects, there are two main stages of categorization: (1) extraction of a general category based on context and its integration with feature binding information at the second level; and (2) precise categorization resulting in explicit object identification at the third level.

In sum, taken together, we suggest that object integration takes place at the second level, and that precise categorization should be considered as part of third-level processing. We agree with the three models that feature integration takes place at the second level, so that a representation of a coherent object from a specific point of view is achieved at this intermediate level. In addition, this representation of a coherent object is integrated with a general category through an initial classification processes. However, integration of objects with their precise category takes place at the third level, which contains viewpoint-invariant representations of objects with their identification.

## Phenomenal Consciousness, Access Consciousness and Perceptual Integration

### Two Notions of Consciousness—The Overflow Debate

There are two central notions of consciousness that are relevant to perceptual consciousness: phenomenal consciousness and access consciousness. Phenomenal consciousness is the experience one undergoes when, e.g., perceiving the world (Block, 2002). Hereafter, “phenomenal consciousness” and “perceptual experience” (or simply “experience”) will be used interchangeably. Phenomenally conscious states involve subjective experiential aspects, so that there is something “it is like” to undergo them. As was emphasized in the previous section, perceptual integration is a key aspect of experience. Access consciousness (or cognitive access) is obtained when a representation can be used freely in reasoning, decision making, report and rational control of action, in virtue of being accessible to WM or to the global workspace (e.g., Dehaene and Naccache, 2001; Baars, 2005; Block, 2008).

Clearly, these are two different concepts of consciousness: the one thoroughly phenomenal and the other thoroughly functional (Block, 2002). However, the fact that the concepts are different does not entail that the phenomena are different. There is a debate regarding whether phenomenal consciousness and access consciousness are two aspects of the same phenomena or whether they are dissociable. According to one view, access consciousness is necessary for phenomenal consciousness (e.g., Dehaene and Naccache, 2001). That is, only representations that are accessible to the capacity-limited WM (3–4 items, see, e.g., Luck and Vogel, 1997) are experienced. This view may seem intuitive, since it reflects a folk psychological tendency of subjects to withhold attributions of consciousness to themselves in cases in which they cannot report, or deliberately act upon, perceptual stimuli. According to the opposite stance, cognitive access is not necessary for phenomenal consciousness—i.e., there are phenomenally conscious states that are inaccessible and thus cannot be reported (Block, 2002, 2007, 2008; Lamme, 2010).

The question whether cognitive access is necessary for phenomenal consciousness is essentially related to another issue regarding the nature of experience: is our experience rich or sparse? Introspection seems to reveal rich perceptual experiences of a world full of colors, shapes and many details. However, phenomena such as change blindness and inattentional blindness appear to suggest otherwise: when viewing a visual scene, subjects fail to notice a change or an appearance of a new salient object, such as a gorilla among basketball players (Block, 2008; Smithies, 2011). Does this mean that we suffer from a radical illusion, so that although it seems to us that our experience is rich, it is in fact sparse? Those who claim that cognitive access is necessary for phenomenality would be inclined to answer that our experience is sparse, as we can only experience up to 3–4 items (in accordance with WM capacity limits), or at most up to 4 detailed representations and several other fragmented or generic representations (Block, 2011). This conclusion is hard to swallow, given that phenomenal consciousness is defined as what it is like for the subject to be in a conscious state, and subjects claim that they experience (in detail) more than they can report.

In an influential paper, Block (2007, 2008) argued that phenomenal consciousness and access consciousness rely on two different memory mechanisms with different capacities. Block agrees that access consciousness relies on WM and is thus limited to 3–4 items. However, he suggests that underlying phenomenal consciousness is a different mechanism—perceptual memory—which has a larger capacity. In one of the key moves of his overflow argument, Block appeals to the work of Landman et al. (2003), which demonstrates that when a retro-cue that indicates a specific item appears after the offset of a presentation of a memory array in a Sperling-like change detection task, subjects are able to detect changes in six to seven items, exceeding the limits of WM capacity. This effect is attributed to storage of detailed representations in a temporary memory mechanism with larger capacity than the capacity of WM—fragile short-term memory (Sligte et al., 2008; Block, 2011; Pinto et al., 2013). According to Block, this storage consists of phenomenally conscious representations, whereas the few representations that are subsequently selected to enter WM are also cognitively accessed. Thus, the capacity of the mechanism underlying phenomenal consciousness “overflows” the capacity of the mechanism underlying access consciousness. Block concludes that since phenomenal consciousness and access consciousness rely on different mechanisms with different capacities and properties, they are separate and dissociable. Opponents of his view claim that although there are some representations that are not accessed in WM, these are not phenomenally conscious (e.g., Cohen and Dennett, 2011).

In the context of the overflow debate, it may be helpful to revisit the assumption that the second level is the locale of the *content* of experience. As already mentioned, all three models presented in the previous section (as well as some others, e.g., Block’s 2008) seem to agree on this assumption. Yet, it should be noted that it is not universally accepted (e.g., Kouider et al., 2010; Cohen and Dennett, 2011). The question what renders a state phenomenally conscious and the question what sorts of contents are phenomenally conscious are considered by many (if not most) philosophers to be separate questions. Nevertheless, the dissociation view has some commitments *vis a vis* the latter question—clearly, it presupposes that the contents of second-level representations are of a sort that can be made phenomenally conscious. As is demonstrated by Prinz’s (2011a) AIR theory, that view may still require further modulation of such representations (that does not amount to their being encoded in WM) in order for them to be phenomenally conscious. Also, it is worth noting that the dissociation view is compatible with the idea that further third-level processing may affect, and specifically add to, the contents of experience—it need not insist that those contents are *exhausted* by the contents of second-level representations. In line with the three models, and as proponents of the dissociation view, we hereafter assume that the contents of second-level representations are of a sort that can be made phenomenally conscious, and further, as proponents of ILH, we adhere to the stronger assumption that the contents of experiences are exhaustively determined by representations of this sort (which can nevertheless be influenced top-down by processing at the third level).

### The Tripartite Models in Relation to the Overflow Debate

Let us consider the relations between the different stances towards the overflow debate and the three models of perceptual processing outlined in Section Levels of perceptual processing: Examination of Ben Shalom’s model. Although not all advocates of these models explicitly allude to that debate, they would agree that access consciousness resides at the third level, because the mechanism underlying this level in all models is WM. They would further agree on the notion that the content of experience (phenomenal consciousness) arises at the intermediate level. Yet, it seems that they would disagree over whether to ascribe phenomenal consciousness to second-level representations or to third-level representations. Specifically, they give different predictions as to whether representations that (due to, e.g., attentional selection limitations) are processed only up to the second level and do not enter the third level (i.e., WM), are phenomenally conscious. Lamme is one of the prominent advocates of the dissociation between phenomenal consciousness and access consciousness. Prinz’ AIR theory stands somewhere in between the two stances. Ben Shalom does not explicitly address the question.

Lamme (2004, 2006, 2010) provides further support for the dissociation between phenomenal consciousness and cognitive access through his neural argument (which was outlined above). According to this argument, recall, there is a fundamental difference in processing between the first and the second levels, since recurrent processing enables integration, learning and plasticity, whereas feedforward sweeps do not. On the other hand, there is no fundamental neural difference between the second and third levels: both allow the key feature of integration through recurrent processing. Lamme provides evidence that processing at the second level is characterized by the important properties we attribute to experience: feature integration, perceptual competition and susceptibility to illusion (Lamme, 2010). The difference between the two levels lies in the extent of recurrent processing, with superficial recurrent processing at the second level and widespread recurrent processing at the third. The spread of activity at the third level enables connections between modules in the global workspace (Dehaene and Naccache, 2001; Lamme, 2010) and thus reportability. However, the *nature* of neural activity is equivalent in the two stages. Therefore, since the only categorical difference is in reportability, there is no reason to ascribe consciousness to the third level but not to the second level.

Contrary to Lamme, Prinz (2011a) argues in his AIR theory (which expands on ILH) that in order for a second-level representation to become conscious, it should be modulated by attention and thus become accessible to WM. Yet, according to Prinz, phenomenality does not require actual access to, or encoding in, WM. Furthermore, representations *in WM* are third-level representations, and as such, according to the ILH component of AIR, they are of a different sort than phenomenally conscious representations: it is second-level representations, rather than third-level ones, that are representations of coherent bound-together objects, including their features represented from a specific point of view. In this sense, then, phenomenal consciousness does overflow the content of WM.

## Access and Phenomenal Consciousness in Autism Spectrum Disorders

### The Relevance of the Overflow Debate for Understanding Consciousness in Autism

Ben Shalom (2005, 2009) suggests that individuals with ASD and TD individuals differ in perceptual integration at the second level. According to her account, people with autism do not have the same basic experiences—the same phenomenally conscious states—as other people do. As we have argued above, integration is considered a key aspect of phenomenal consciousness, and thus lack of typical integration in autism would seem to imply different phenomenology.

Notwithstanding, the debate regarding whether phenomenal consciousness and access consciousness are dissociable has implications for the understanding of experience in autism. If access consciousness is necessary for phenomenal consciousness, then, given that people with ASD have lesser access to integrative information, the conclusion that phenomenal consciousness is atypical in autism is inevitable. However, if cognitive access and phenomenal consciousness are dissociable, then another possibility emerges: it is possible that the apparent atypicality in integration among individuals with ASD stems from atypical accessibility, rather than atypical experience. That is, it is possible that integration at the second level is similar among people with and without ASD, but the difference lies in integrative information at the third level. Specifically, we suggest that individuals with ASD have typical phenomenal experiences of objects, yet access those experiences differently: although viewpoint-specific representations of perceptual objects are achieved at the second level among both autistics and non-autistics, viewpoint-invariant representations and precise categorization at the third-level are accessible to a lesser extent among individuals with autism (be it due to less accessibility of these representations or lack of automatic formation of such representations). Here, we suggest a framework in which a core characterization of ASD lies in atypicality at the stage in which perceptual representations of objects (formed at the second level) are integrated with precise categories, yet the experiences of individuals with ASD and those of TD individuals are similar.

Key is the need for a clearer specification of the type of atypical integration in ASD. There is little doubt that the most basic sort of integration, namely feature binding, is achieved in ASD (e.g., Plaisted et al., 1998; see Section Evidence for typical second-level integration in autism). Yet, this leaves room for two possible notions of atypicality: insufficient emphasis of the representation of binding (e.g., the conjunction “white and round”) or lack of precise categorization (e.g., “plate”; Ben Shalom, 2005). Both notions are consistent with the possibility that integration at the second level among individuals with ASD is typical, and that the abnormality stems from inaccessibility of integrated representations. According to the first possibility, lack of emphasis of the binding information, manifested in lower activation of binding information, results in the representations’ failing to be selected and mobilized to the third level. With regard to the second possibility, if the locale of higher categorization is considered to be at the third (rather than the second) level (as is the case, e.g., according to ILH and 2SI, and as we suggested in Section The importance of integration for experience), then atypicality lies in difficulty to assign the object to the appropriate category at the third level, due to lesser accessibility of viewpoint-invariant representations of objects. Ben Shalom’s suggestion that lack of integration in ASD is compensated by effortful use of third-level resources is consistent with this interpretation.

In the following section, we review findings regarding integration and object categorization among people with ASD and provide evidence indicating typical second-level representation and atypical third-level representations in autism. We suggest that the second option of atypical integration in autism is most suitable for explaining the data, i.e., that atypicality in integration in autism consists in lack of precise categorization. Hereafter, our examination of the nature of representations among individuals with and without autism relies mostly on Prinz’ characterization of representations at different levels of processing, and our examination of neural activity among autistics and non-autistics relies mostly on Lamme’s framework.

### Evidence for Typical Second-Level Integration in Autism

As mentioned, there is little doubt that individuals with ASD achieve feature binding, and thus possess representations of coherent objects. First, people with ASD detect conjunctions of features in the visual search paradigm even faster and more accurately than TD individuals (Plaisted et al., 1998; O’Riordan and Plaisted, 2001; O’Riordan et al., 2001; O’riordan, 2004). In the conjunction condition of the visual search, targets and distractors contain different combinations of similar features, and thus performance on this task would be impossible without feature binding. Furthermore, TD individuals and individuals with ASD perform similarly on discrimination tasks that require color and shape conjunctions (Plaisted et al., 2003). In the discrimination and the visual search tasks, subjects with ASD do not have a conjunction cost: they perform similarly when asked to detect a target according to either a feature or a combination of features (Plaisted et al., 1998, 2003). In addition, children with ASD outperform TD children in mental rotation tasks as well (Falter et al., 2008), which require representations of objects as wholes. Finally, individuals with ASD seem to form “event files” (Zmigrod et al., 2013)—integrated representations of associations between objects and behavioral responses. Event file formation is indicated by an implicit measure of repetition cost—a performance deficit that is evident after incomplete repetitions of stimulus-feature or stimulus-response combinations, as compared to complete repetition or alternation. All these findings indicate that feature binding, and thus the formation of representations of objects, is intact in autism. Moreover, such data refute the possibility that binding information is not accessible to individuals with ASD. On the contrary, it suggests that people with autism have an even better access to representations of this type.

We should note that according to O’Riordan and Plaisted (2001; O’riordan, 2004), the superiority of autistics in visual search does not stem from better feature integration, but rather from an enhanced ability to discriminate between similar items (e.g., targets and distractors). In support of this claim, they found that when performing triple conjunctions conditions (i.e., the target is identified using three features) compared to conjunctions of two features, and when features were highly similar in featural dimensions, subjects with ASD had less cost compared to TD participants.

Susceptibility to visual illusions is another manifestation of a sort of integration that is considered an aspect of phenomenal consciousness (Lamme, 2010). Visual illusions have a remarkably strong impact on experience: TD individuals report experiencing them even when they know how these illusions affect perception (e.g., Bruno and Franz, 2009). Susceptibility to illusions should take place at the second level of processing (Lamme, 2010), and it requires the presence of a representation of an integrated object. Ropar and Mitchell (1999) examined susceptibility to visual illusions such as the Titchener circles and the Muller-Lyer figures, among subjects with ASD. They used two measures in order to assess both explicit and implicit susceptibility to illusions. Explicit susceptibility was assessed using verbal judgment of the stimuli (e.g., subjects were asked “are these two circles different sizes or the same size?” with regard to a Titchener circles stimulus). In the implicit measure, participants were asked to adjust the lengths of lines or size of circles rather than judge them explicitly. Subjects with ASD and TD subjects did not differ in their performance in both versions of the task. Garner and Hamilton (2001) reported susceptibility to visual illusions among subjects with autism as well. We should note that Happé (1996) failed to find susceptibility to illusions among subjects with ASD. However, Happé’s results may have been influenced by other factors (see Ropar and Mitchell for criticism of Happé’s methodology). Thus, most evidence indicates that individuals with ASD are susceptible to visual illusions similarly to TD people, and may also access the experience of these illusions.

In addition, autistics seem to have typical use of contextual information. López and Leekam (2003) found that participants with and without ASD identified objects presented after a contextually-appropriate scene (e.g., a kettle presented after a presentation of a kitchen scene) faster and more accurately than objects presented after a contextually-inappropriate scene (e.g., a football presented after a presentation of a kitchen scene). That is, object identification was similarly facilitated by the appropriate context in both TD individuals and individuals with ASD. These findings indicate typical use of context in object recognition, a process that according to Bar (2004) occurs early in the hierarchy and that Ben Shalom includes at the second level. It should be noted that López and Leekam’s results revealed that subjects with ASD made more errors in naming objects, regardless of whether the previous scene provided appropriate context or not, a finding that we suggest may point toward a third-level deficit in precise categorization, rather than atypicality in second-level integration.

In sum, central properties of perceptual integration that, according to the evidence presented, characterize second-order representations—feature integration, susceptibility to visual illusions and use of context in aiding object recognition—seem to be similar among TD and ASD individuals; and to the best of our knowledge, there is no evidence suggesting atypicalities in second-level representations in ASD. We conclude that it is plausible that perceptual integration characteristic of the second level is not atypical among people with ASD.

### Evidence for Atypical Third-Level Integration in Autism

Studies such as those reviewed in the previous section have led to modifications of the weak central coherence theory (Frith and Happé, 1994). Originally, the theory postulated that there is a core failure in global processing in autism that leads to a reduced ability to integrate component features of a figure into a coherent whole. Later, Happé and Frith (2006) revised the theory, and put more emphasis on superior local processing as reflecting a cognitive style. Plaisted (2001) suggests a competing hypothesis, the reduced generalization theory, according to which there is reduced processing of similarities between objects in autism, and yet processing of differentiating or unique features is intact. This theory postulates that individuals with autism should show lower categorization abilities, which we suggest in the present section is to be attributed to an atypicality in third-level processing, rather than to an anomalous integration at the second level. The reduced generalization and the weak central coherence theories are consistent with both conceptions of atypicality in integration we have suggested—insufficient emphasis of the representation of binding information or a deficiency in precise categorization. However, since the tasks reviewed above indicate high accessibility of representations of binding, the second possibility is more plausible. In this section we examine the second conception of atypicality, i.e., the one on which there is a deficit in higher categorization among individuals with autism. We provide evidence for atypicality in third-level processing among autistics, manifesting in divergence in properties of third-level representations and neural activity.

#### Evidence for Viewpoint Variant Third-Level Representations in Autism

We have argued that representations of coherent objects matching second-level representations seem to be typical among individuals with ASD. However, we found that there are some indications of atypicality in tasks that require third-level processing. Specifically, we suggest that there is some evidence for atypical formation of viewpoint-invariant object representations at the third level (as it is conceptualized by ILH) among individuals with autism—such representations are not as easily and automatically formed in ASD.

Ropar and Mitchell (2002) examined shape constancy in autism. They showed participants images of ellipses in three conditions, and then asked them to recreate the shape they saw on a different screen. In the prior knowledge condition, the shape was presented alone in a darkened display without any perspective cues, and participants were told that the shape is a slanted circle. In the prior knowledge and perspective cues condition, the shape was presented in an illuminated display containing perspective cues and participants were told the shape presented is a slanted circle. In the control condition, the shape presented was a non-slanted ellipse, presented without any perspective cues. All participants recreated the shape as more circular in the prior knowledge and prior knowledge and perspective cues conditions compared to the control condition. TD participants and participants with ASD differed only in the prior knowledge condition: participants in the ASD group exaggerated circularity less than TD participant, and recreated a shape that more closely resembles the original one. This task examines WM representations of objects, because participants had to recreate the shapes based on their memory. In ILH terms, it seems that individuals with ASD had more difficulty creating a viewpoint-invariant representation of the circle when they were provided with prior knowledge regarding the identity of the shape, thus relying more on the representation of the perceptual object created at the second level (an ellipse), which is dependent on viewpoint. On the other hand, TD individuals relied on a viewpoint-invariant representation (a circle) in accordance with prior knowledge. When perspective cues were provided, individuals with ASD perceived the object as more circular, similarly to TD individuals. In this case, since cues were apparent, representations at the second and third levels should be more similar.

Mottron et al. (1999) found that individuals with autism were better than TD individuals in reproducing impossible 3D figures. Subjects with ASD copied the figures faster than TD participants. In addition, while TD subjects copied impossible figures considerably slower than possible figures, there was a smaller cost of impossibility among subjects with ASD. These findings have recently been replicated (Sheppard et al., 2009). Copying involves the construction and maintenance of a representation of the object in visual WM (Guérin et al., 1999), and thus performance relies on third-level representations. Mottron et al. explain their results as emerging from a difficulty relating elements of a figure among individuals with ASD, perhaps due to limited capacity to hold parts of objects in WM simultaneously. We suggest that, similarly to our interpretation of Ropar and Mitchell’s (2002) results, third-level representation among individuals with ASD are more similar to second-level representations, in that they are viewpoint specific, rather than viewpoint invariant. First, this task requires maintaining the shape in WM while copying and thus reflects representations held in WM. In addition, the impossibility of the figure should manifest mainly at the third level of the hierarchy: at the second level, the drawing should be represented as it is seen (from the viewpoint of the observer), yet the fact that impossible figures violate 3D rules obstructs formation of a viewpoint-invariant representation at the third level. The superiority of individuals with ASD may thus reflect differences in third-level representations.

Liu et al. (2011) found that in an explicit possibility task, in which participants were asked to judge whether figures are impossible or not, participants with and without ASD had comparable performance and similar patterns of brain activation. During this task, both TD subjects and subjects with ASD recruited more frontoparietal areas compared to a second task, in which the same stimuli were used but participants were not required to judge the possibility of the figures. However, in the second task participants with ASD showed less interference from impossible figures, as well as less activation in medial-frontal areas and less connectivity between medial-frontal and posterior-visuospatial areas compared to TD participants. Liu et al. explain their results as indicating a greater need to suppress the global configuration of the figure in the TD group, as indicated by higher activation in frontal areas related to high-level executive functions (such as medial and superior frontal regions). These results may indicate that third-level representations in the form of viewpoint-invariant representations of objects can be achieved among individuals with ASD, but these are not achieved automatically. That is, whereas representation among TD individuals become viewpoint-invariant automatically once they are processed at the third level, among individuals with ASD third-level representations are more similar to second-level representations (i.e., viewpoint-specific), but can be processed to become viewpoint-invariant according to need (e.g., task demands). This explanation is consistent with Ben Shalom’s suggestion that individuals with autism can overcome their difficulty in integration via use of effortful logical processing of perception. In addition, our explanation is in agreement with Mottron et al.’s (2006) claim that high-level processing is automatic or mandatory among TD individuals, but optional among individuals with autism.

Another image copying study, using bistable ambiguous figures such as the duck/rabbit image, reveals viewpoint specific third-level representations in autism as well. Allen and Chambers (2011) examined implicit and explicit processing of ambiguous figures among participants with ASD who had cognitive delay and children with intellectual disability. First, participants were asked to copy ambiguous and non-ambiguous figures, after either being given a label for a presented figure (e.g., “draw this rabbit”) or not (“draw this picture”). Afterwards, participants were asked several times what they see in the image, in order to assess spontaneous reversals. Then, participants who did not report perceiving the alternative interpretation of the figure were informed about it and informed reversals were assessed. Finally, they were asked to copy the image once again. Results indicate that when participants were not given a label before they drew the pictures, both groups provided similar figures before and after reversals. However, the groups were affected differently by the introduction of a label: participants in the learning disability group provided different drawings after a label compared to the first drawing (indicating that labeling one interpretation influenced their construction of the drawing), whereas participants in the ASD group provided two similar drawings regardless of the label. This may indicate, once again, that individuals with autism have third-level representations that are more similar to second-level representations. The two groups had comparable explicit interpretation reversals, and in both groups the number of reversals increased after participants were informed about the bistability of the figures. This suggests that effortful use of reasoning resources is typical in autism, consistently with Ben Shalom’s claim that individuals with ASD compensate for lack of integration by using effortful reasoning, and with Mottron et al.’s suggestion that high-level processing can be achieved at will in autism. Allen and Chambers concluded that individuals with autism have typical perceptual representations but atypical conceptual representations. This conclusion is in line with our suggestion that third-level, rather than second-level, representations are atypical in autism.

#### Evidence for Atypical Use of Third-Level Precise Categorization in Autism

According to Marr (1982) and the ILH, third-level representations are important for categorization and conceptual processing. Thus, atypicality in viewpoint-invariant representations is expected to have implications for categorization. In her reduced generalization theory, Plaisted (2001) suggests that among people with autism discriminating features are more salient, while features held in common between objects are processed to a lesser degree, giving rise to atypicality in formation of categories and concepts. The ability to generalize across situations is reduced due to saliency of differentiating aspects of separate cases. Her account is supported by a personal description by Grandin:

“…my concept of dogs is inextricably linked to every dog I’ve ever known. It’s as if I have a card catalogue of dogs I have seen, complete with pictures …” (Grandin, 1996, p. 28).

O’riordan (2004) has added that generalization capacities are important for high-level processes, such as categorization and reasoning. Consistent with the reduced generalization theory, Church et al. (2010) found that high-functioning autistics use family resemblance (or a family prototype) less than controls during categorization of objects (e.g., when judging whether a non-social figure belongs to a newly learned category). Plaisted’s explanation appeals to third-level integration atypicality. Thus, the reduced generalization theory is compatible with our suggestion that atypicality in integration among people with ASD is at the third level.

The change detection paradigm is one of the most commonly used in the study of perceptual consciousness. In this paradigm, subjects are presented with two pictures of a scene that may differ in one detail (e.g., one item has changed its color or disappeared), and are asked to detect the change. Findings consistently reveal a phenomenon called “change blindness”: people fail to notice the change, even after re-viewing both pictures several times. The prevalent explanation is that changes in items to which attention is not allocated cannot be detected. One must compare the original image, stored in WM, with the new image, and thus an item must reach WM in order for change to be recognized. Some infer from this phenomenon that even though we think we have a rich experience of the world, we in fact have a sparse experience (e.g., O’Regan and Noë, 2001). This stance is usually supported by those who advocate the view that access consciousness and phenomenal consciousness are not dissociable. Others explain change blindness as stemming from failure in accessibility (e.g., Block, 2001; Prinz, 2011a; Smithies, 2011), so that the changed object is experienced, yet the change is not detected due to deficiency in its storage in WM. According to Block (2001, 2008), this lack of accessibility is a result of failure in conceptualization of the changed item.

Several studies have examined differences in change blindness between TD individuals and individuals with autism. When change is perceptual and does not require precise categorization, people with ASD seem to perform similarly to TD people. For example, Burack et al. (2009) found similar accuracy and reaction times among subjects with ASD and TD subjects who were asked to indicate which of two images of objects presented simultaneously side by side has changed.^2^ Since the objects depicted in their study were presented without a background scene, detection of changes could have been executed based on perception, without a demand to categorize the objects. Nonetheless, other studies, in which manipulations of context and semantic role were introduced, indicate impairments in processing of precise categories, although these results are not conclusive.

With regard to contextual influences on detection of changes in a scene, Loth et al. (2008) demonstrated that individuals with autism are less sensitive to influences of context. Performance of subjects in the TD and ASD groups was similar when changes were contextually-appropriate (either from the same general category, e.g., replacement of a kettle with a frying pan, or from the same precise category, e.g., replacement of a kettle with a different one). However, the groups differed when changes were contextually-inappropriate (e.g., replacement of a kettle in a kitchen with a football): While TD individuals detected changes in this condition faster than in contextually-appropriate conditions, individuals with ASD had similar performance in all change conditions. Contrary to Loth et al., Fletcher-Watson et al. (2006) found a similar pattern of results among subjects with ASD and TD subjects, and that both groups detected contextually-inappropriate changes faster than they detected contextually-appropriate changes. However, in Fletcher-Watson et al.’s version of the task, participants could control switches between the two images, so that they could choose when to look back at the original scene, and participants with ASD made more switches compared to TD participants. This methodological difference may account for the discrepancies in the results of the two studies.

This lack of benefit in detection of contextually-inappropriate stimuli may indicate a third-level deficit in categorization. Note that the kind of context that models such as Bar and colleagues’ (Bar, 2004; Bar et al., 2006) and the 2SI model (Schendan and Kutas, 2007; Schendan and Stern, 2008) refer to is not relevant in this case, because in the case of contextually-inappropriate items, in which changes are detected more easily among TD individuals, early top-down contextual effects should not aid object recognition (on the contrary, these models will predict slower processing of these objects, see Bar, 2004). In Loth et al.’s (2008) and Fletcher-Watson et al.’s (2006) experiments, participants were provided with prolonged exposure to stimuli, and thus it is more reasonable that inappropriate items were processed bottom-up in a manner more in line with Marr’s levels of processing. As was noted in the previous section, facilitation of object recognition based on context in the sense Bar refers to seems to be typical among individuals with autism (López and Leekam, 2003). The lack of difference between subjects with ASD and TD subjects in contextually-appropriate changes in Loth et al.’s (2008) study supports this conclusion as well.

Semantic processing is yet another kind of integration examined by using the change detection paradigm. Results regarding sensitivity to semantic roles of items in scenes are conflicting as well. Smith and Milne (2009) examined change detection according to the semantic role of items using short films. They found that, overall, subjects with ASD detect continuity changes in the films better than TD subjects. In addition, while participants in both groups detect central changes more accurately than marginal changes, the difference between the conditions is smaller among individuals with ASD, indicating less sensitivity to semantic information. On the other hand, Fletcher-Watson and colleagues (Fletcher-Watson et al., 2006, 2012) used still images of scenes and found that, similarly to TD participants, individuals with ASD detect changes in items with a central semantic role (e.g., a boat on the river) better than changes in items that had a marginal role in the scene (e.g., a tree among other trees). However, they found that participants in the ASD group were slower to detect changes in marginal items compared to participants in the TD group, and made more switches back to the original scene in this condition. Their results, as opposed to Smith and Milne’s, indicate sensitivity to semantic information and greater saliency of semantically central items compared to semantically marginal items. Methodological differences may account for discrepancies in this case as well. Smith and Milne’s task was more demanding: they used a dual task, in which in addition to change detection, participants also had to follow the plot of the scene and answer semantic question regarding what was going on. It could be that the lack of effect of semantic role in the ASD group was caused by high cognitive load on participants’ WM, which could interfere with conceptual processing and effortful integration of precise categories and objects. Thus it could be argued that under low cognitive load, semantic processing can take place and an integrated representation of both the object and its precise category can be achieved, at least with regard to semantically salient objects. However, under high cognitive load or for semantically marginal objects, this integration is not achieved among individuals with ASD. In the case of high load, this could be due to lack of resources required for effortful integration or the creation of viewpoint-invariant representation of the object for identification. In the case of semantically marginal objects this could result from attentional processes. Fletcher-Watson et al. (2006) explained the finding that individuals with ASD identified changes in marginal items slower than TD individuals as reflecting a deficit in attentional shifting between items or away from central items (which were selected first). In line with other findings reviewed above, this may point to a WM deficit: such an explanation involves updating the content of WM, because it requires the removal and substitution of central items with marginal ones.

#### Evidence for Atypical Neural Activity Attributed to the Third Level in Autism

Lamme (2004, 2006, 2010) addresses differences between the second and third levels in terms of patterns of neural activity. Second-level activity is characterized by local recurrent processing between visual areas, whereas third-level activity is more widespread and includes co-activation of frontoparietal areas as well. The literature on neural processing in autism points to atypical global connectivity and functioning of areas related to WM or the global workspace, in accordance with our suggestion of a third-level abnormality among individuals with ASD.

Ring et al. (1999) conducted an fMRI study that examined brain activity of subjects with ASD and TD controls during performance in the embedded figure task. In this task, which is thought to involve visual WM, subjects are presented with a complex pattern and a simple figure, and are asked to identify the hidden simple figure in the complex design. The general finding is that autistics outperform TD subjects in this task (Jolliffe and Baron-Cohen, 1997). Ring et al. found that participants with ASD and TD subjects had a similar pattern of activation in most brain areas. However, the ASD group had higher activation in association areas, whereas the control group had higher activation in frontoparietal areas associated with WM (the global workspace) and attentional allocation: the dorsolateral prefrontal cortex and the superior parietal lobule (Dehaene and Naccache, 2001; Baars, 2005). A comparable pattern of less activation in global workspace areas among subjects with ASD compared to TD subjects and similar activation in other visual processing areas was observed by Luna et al. (2002) as well, using a different spatial WM task. Ring et al. suggest that this pattern may indicate that subjects with ASD use more mental imagery when performing the embedded figure task, while TD participants rely more on their WM. We explain these results as reflecting use of third-level representations that are viewpoint-variant rather than invariant in WM among individuals with autism. This interpretation is supported by Ranganath’s (2006) suggestion that the dorsal prefrontal cortex is in charge of the manipulation and reconstruction of complex stimuli, via reduction of the saliency of relations between features based on prior knowledge. Our explanation is consistent with typical explanations of superiority of individuals with ASD in this task, which appeal to less interference from the global shape and an ability to focus on local details (e.g., Happé and Frith, 2006).

Just et al. (2004) found that during sentence completion tasks there is less connectivity between the dorsolateral prefrontal cortex and parietal and occipital areas among participants with ASD compared to TD subjects. They suggest the underconnectivity theory, according to which autism is caused by lesser functioning of integrative circuitry in frontal integrating centers, especially when tasks require high-level abstraction and during high load. Although the task utilized in their study is not perceptual, and may seem irrelevant to our argument, the underconnectivity revealed in their study involves frontoparietal areas engaged in the global workspace, which is not specific to certain modalities or cognitive domains (Dehaene and Naccache, 2001). Similarly, Courchesne and Pierce (2005) have proposed that in autism there is increased local connectivity between close areas (including increased local frontal activity) and reduced long-distance co-activation between the frontal lobe and other regions. They suggest that this pattern leads to impairments in widespread processing and in integration of information from separate modules and in spreading of contextual feedback towards lower areas. Both descriptions fit impairment at the third-level as conceptualized by Lamme.

## Conclusion and Implications

### The Argument for Locating the Atypicality in Autism at the Third-Level

In line with Ben Shalom’s model and other tripartite-models of perceptual processing (ILH and Lamme’s model), we assume that processing of visual information can be usefully characterized as proceeding in three stages. The models differ with respect to the level at which certain categories are integrated with representations of objects. In agreement with Lamme’s model and the 2SI model, we suggest that initial classification is achieved at the second level, yet objects are ascribed to their precise categories at the third level.

Based on empirical evidence, we have argued that autistics and non-autistics have similar viewpoint-specific second-level representations, characterized by feature binding, susceptibility to illusions and contextual facilitation. However, we suggest that individuals with autism have atypical third-level processing. First, whereas third-level representations in TD individuals seem to be viewpoint-invariant, data from studies examining shape constancy and copying impossible figures and ambiguous figures suggests that individuals with ASD have viewpoint-specific third-level representations, which are more similar to second-level representations. Second, there is some evidence for abnormality in third-level representations in individuals with autism that consists in a deficiency in the integration of perceptual representations of objects with precise categories. (We agree with Ben Shalom that individuals with autism can, at will, make up for the difficulty in categorization via effortful use of WM resources and use of logic and reasoning; they are capable of manipulating their third-level representations so that these would allow object recognition.) Third, individuals with autism seem to have atypical neural activity that is associated with third-level processing: lower global connectivity and lower activation in frontoparietal areas related to WM or to the global workspace in comparison with TD individuals. We conclude that atypicality in integration in autism is better conceptualized as third-level atypicality than as second-level atypicality.

The debate regarding whether phenomenal consciousness and access consciousness are dissociable and our argument for the claim that the central atypicality in autism resides at the level of third-level rather than second-level representations are interconnected. On the one hand, the stance one takes with respect to that debate has implications for one’s interpretation of our framework for understanding the atypicality in autism as a third-level atypicality—specifically, for whether this atypicality is understood as pertaining to phenomenal consciousness or to access consciousness. On the other hand, our argument may have implications for that debate—the commitment of a particular stance to attribute, or withhold attributions of, typical basic phenomenal states to autistics may be useful for evaluating its plausibility.

### Understanding Phenomenal Consciousness in Autism

Different stances in the debate over whether phenomenal consciousness and access consciousness are dissociable may lead to different interpretations of our suggestion that individuals with autism have typical second-level processing but atypical third-level processing. That debate, recall, takes the form of a scientific controversy over whether second-level representations are phenomenally conscious (the dissociation view), or whether phenomenality requires that a representation be modulated by third-level processing, and is therefore restricted to third-level representations. Thus, if access consciousness and phenomenal consciousness are not dissociable, i.e., if the latter requires the former, then regardless of whether the atypicality arises already at the second level or is restricted to the third level, individuals with autism and TD individuals differ in their basic experiences. The non-dissociation view entails that every difference in access consciousness implies a difference in phenomenal consciousness, hence the fact that the access consciousness of autistics is atypical implies that their phenomenal consciousness is atypical as well. However, if the two sorts of consciousness are dissociable, atypicality of access consciousness does not imply atypicality in phenomenal consciousness. In that case, the view for which we have argued—namely, that the abnormality characteristic of autism is restricted to the third-level and that second-level processing and representations in autistics are intact—opens the door for the possibility that TD individuals and ASD individuals share their basic experiences. Due to our adherence to the dissociation view, it is this latter possibility that we endorse.

It should be noted that, even granted our claim that the atypicality in autism is located at the third-level, there is a possible middle position regarding the phenomenal states of individuals with autism. This position follows if one holds that second-level representations are phenomenally conscious, yet rejects the assumption (which we tend to endorse) that the contents of phenomenal states are *exhaustively* determined at the second level. That assumption is rejected by those who take the contents of perceptual phenomenal states to be (at least partly) conceptual, or, relatedly, to represent not only “simple” properties such as color, shape, illumination and motion, but also more “sophisticated” properties, such as plate, tree or eucalyptus (see, e.g., Siegel, 2006, 2010). Their view may still be compatible with the dissociation of phenomenal consciousness and access consciousness, provided that they hold that the contents of phenomenal states of TD individuals who are conceptually sophisticated typically involves two layers, the first of which is derived from the second-level and the second of which is derived from the third level. Conjoined with our claim that the atypicality in autism characterizes the third-level, that view would predict that the experiences of autistics have a lot in common with those of TD individuals, yet there are also some differences, due to difficulties autistics have applying certain specific categories at the third level.

### Implications of the Case of Autism for the Overflow Debate

An important question for any account of phenomenal consciousness concerns the plausibility of its commitments regarding ascriptions of various phenomenally conscious states. That is, the question concerns the set of subjects (or creatures) to whom the account attributes any phenomenality, and (what is more relevant for our purposes) to whom it attributes phenomenality that is similar to that of typical (mature, human) subjects. Put in prevalent philosophical jargon, an account that is committed to attributions of phenomenal states similar to those of typical subjects to subjects who seem to lack them is said to be (too) liberal; an account that is committed to withholding such attributions from subjects who seem to have them is said to be chauvinist. Clearly, our pre-theoretic judgments regarding attributions of phenomenally conscious states form only a fallible starting point, which may be overturned by scientific and theoretical investigations. Yet, other things being equal, it is an advantage of a theory of mentality if it matches those judgments—we should strive for a reflective equilibrium between our pre-theoretic judgments on the one hand, and considerations regarding e.g., the explanatory powers of particular theories of phenomenality on the other hand.

A clear example that concerns ASD is the charge of chauvinism made by many philosophers against central versions of the higher-order thought (HOT) theory of consciousness. According to this theory, “a mental state is a conscious state when, and only when, it is accompanied by a suitable HOT” (Rosenthal, 1990/1997, p. 741), or, more specifically, if it is accompanied by a thought—grounded non-inferentially and non-observationally—to the effect that one is in that state. Autism is of course associated with theory of mind deficits, and specifically, individuals with autism have been claimed to have impaired ability to form HOTs about their experiences (see, e.g., Perner, 1998; Frith and Happé, 1999). Given that at least some low-functioning autistics cannot form any such thoughts, it follows from HOT that such autistics are not phenomenally conscious! While some (e.g., Perner, 1998; Carruthers, 2000) have bitten that bullet and accepted that consequence, it is considered by others to be a *reductio ad absurdum* of HOT.

Now, we by no means suggest that the charge of chauvinism that can be mounted against the non-dissociation view of the relations between phenomenal consciousness and access consciousness is as strong as the charge just described against the HOT. The non-dissociation view is not committed to denying that individuals with ASD are phenomenally conscious; it is committed only to their having quite atypical phenomenal states. Nevertheless, it seems to us that the non-dissociation view tends to exaggerate the differences between the mental lives of autistics and TD individuals. No doubt, there are significant differences. We believe that the dissociation view adequately accounts for those differences by taking them to be differences in access consciousness (rather than in phenomenal consciousness) i.e., by taking the bulk part of the atypicality in autism to pertain to the ways autistics access their experiences. The non-dissociation view, in contrast, is committed to interpreting the data described in this paper regarding those aspects of perceptual processing that are shared among subjects of the two groups as having little relevance to the personal level, and as scarcely reflected in the most basic ways in which they experience the world. This consequence of the non-dissociation view seems to us less plausible, and so as one that, other things being equal, should count against it.^3^

## Conflict of Interest Statement

The Review Editor Hagit Benbaji declares that, despite being affiliated to the same institution as authors Tal Yatziv and Hilla Jacobson, the review process was handled objectively and no conflict of interest exists. The authors declare that the research was conducted in the absence of any commercial or financial relationships that could be construed as a potential conflict of interest.

## Footnotes

^1^Lamme has recently divided this level into two distinct levels—superficial feedforward sweeps, which are limited to visual areas, and deep feedforward sweeps, which reach the prefrontal cortex (Lamme, 2010). This distinction leads him to claim that consciousness and attention are orthogonal. This issue is less relevant to our present discussion, and therefore we will focus on the original and simpler version of his distinction.

^2^It should be noted that in the TD group mental age was correlated with performance, whereas in the ASD group there was no such correlation, a result that may indicate different developmental trajectories. However, overall detection of changes in objects was similar in the two groups.

^3^The charge of chauvinism against the non-dissociation view can be generalized so as to apply to its commitments regarding the phenomenal states of non-conceptual human subjects and non-human animals. For criticisms, in the same spirit, against the view that the contents of the experiences of mature humans are thoroughly conceptual—i.e., ones according to which that view overstates the differences between the sort of perceptual awareness enjoyed by mature humans on the one hand and non-conceptual creatures on the other hand—see, e.g., Jacobson and Putnam, 2015; Collins, 1998 and Ayers, 2004 (for weaker accusations along similar lines, see,e.g., Wright, 2002; Burge, 2003). For a defense of the claim that the dissociation view is compatible with the claim that phenomenal states are “of the subject” and belong to the personal level, see Jacobson (2014).
